# Multiparametric vs. Inferior Vena Cava–Based Estimation of Right Atrial Pressure

**DOI:** 10.3389/fcvm.2021.632302

**Published:** 2021-03-08

**Authors:** Matteo Toma, Stefano Giovinazzo, Gabriele Crimi, Giovanni Masoero, Manrico Balbi, Fabrizio Montecucco, Marco Canepa, Italo Porto, Pietro Ameri

**Affiliations:** ^1^Cardiovascular Disease Unit, Istituto di Ricerca e Cura a Carattere Scientifico Ospedale Policlinico San Martino, IRCCS Italian Cardiology Network, Genova, Italy; ^2^Department of Internal Medicine, University of Genova, Genova, Italy; ^3^First Clinic of Internal Medicine, IRCCS Ospedale Policlinico San Martino, IRCCS Italian Cardiology Network, Genova, Italy

**Keywords:** right atrial pressure, echocardiograghy, right heart catheterization, heart failure, pulmonary hypertension

## Abstract

**Background:** Right atrial pressure (RAP) can be estimated by echocardiography from inferior vena cava diameter and collapsibility (eRAP_IVC_), tricuspid *E*/*e*′ ratio (${\text{eRAP}}_{\text{E}/{\text{e}}^{\prime }}$), or hepatic vein flow (eRAP_HV_). The mean of these estimates (eRAP_mean_) might be more accurate than single assessments.

**Methods and Results:** eRAP_IVC_, ${\text{eRAP}}_{\text{E}/{\text{e}}^{\prime }}$, eRAP_HV_ (categorized in 5, 10, 15, or 20 mmHg), eRAP_mean_ (continuous values) and invasive RAP (iRAP) were obtained in 43 consecutive patients undergoing right heart catheterization [median age 69 (58–75) years, 49% males]. There was a positive correlation between eRAP_mean_ and iRAP (Spearman test *r* = 0.66, *P* < 0.001), with Bland–Altman test showing the best agreement for values <10 mmHg. There was also a trend for decreased concordance between eRAP_IVC_, ${\text{eRAP}}_{\text{E}/{\text{e}}^{\prime }}$, eRAP_HV_, and iRAP across the 5- to 20-mmHg categories, and iRAP was significantly different from ${\text{eRAP}}_{\text{E}/{\text{e}}^{\prime }}$ and eRAP_HV_ for the 20-mmHg category (Wilcoxon signed-rank test *P* = 0.02 and *P* < 0.001, respectively). The areas under the curve in predicting iRAP were nonsignificantly better for eRAP_mean_ than for eRAP_IVC_ at both 5-mmHg [0.64, 95% confidence interval (CI) 0.49–0.80 vs. 0.70, 95% CI 0.53–0.87; Wald test *P* = 0.41] and 10-mmHg (0.76, 95% CI 0.60–0.92 vs. 0.81, 95% CI 0.67–0.96; *P* = 0.43) thresholds.

**Conclusions:** Our data suggest that multiparametric eRAP_mean_ does not provide advantage over eRAP_IVC_, despite being more complex and time-consuming.

## Introduction

Right atrial pressure (RAP) is an important prognostic factor in pulmonary hypertension (PH), regardless of whether this latter is due to pulmonary vascular disease, especially pulmonary arterial hypertension (PAH), or heart failure (HF) (1–3).

RAP estimation (eRAP) is usually performed by echocardiography, by assigning a value on a 5-mmHg scale based on inferior vena cava (IVC) diameter and respiratory variation (eRAP_IVC_) (4–6). Alternatively, RAP may be estimated by assessing the tricuspid *E*/*e*′ ratio (${\text{eRAP}}_{\text{E}/{\text{e}}^{\prime }}$) or by analyzing the hepatic vein (HV) pulsed wave (PW) Doppler spectra (eRAP_HV_) (4, 7–10). All these approaches have limited accuracy (11–13), and it has recently been suggested that the mean of eRAP_IVC_, ${\text{eRAP}}_{\text{E}/{\text{e}}^{\prime },}$ and eRAP_HV_ (eRAP_mean_) is more accurate than eRAP_IVC_ (14). However, this method has been tested only in patients with a left ventricular assist device (LVAD) (14).

The scope of this study was to investigate the correlation between eRAP_mean_ and its components, including eRAP_IVC_, and invasively measured RAP (iRAP) in a cohort of subjects undergoing right heart catheterization (RHC) for different reasons.

## Methods

### Study Population

In this prospective, observational, single-center study, we consecutively enrolled the patients who underwent RHC between September 2018 and January 2020 and had at least two components of eRAP_mean_ measurements. For subjects undergoing multiple RHC during the study period, only the first one was considered. As per institutional policy on admission, all patients signed an informed consent to the use of their anonymized clinical data for research purposes. The study protocol was conducted in accordance with the ethical guidelines of the 1975 Declaration of Helsinki.

### Echocardiography

A two-dimensional transthoracic echocardiogram was performed by two cardiologists (M.T. and S.G.) blinded to the results of RHC, on the same day of the hemodynamic assessment.

Standard images were acquired with the patient in the lateral decubitus position. Left ventricular (LV) dimensions and function were evaluated in the parasternal long-axis and apical four-chamber views. Mitral and aortic regurgitations were evaluated using color Doppler and continuous-wave Doppler in the apical four- and five-chamber views. LV diastolic function was examined through PW Doppler of the transmitral flow (E-wave and A-wave peak velocities, E/A ratio, deceleration time of the E-wave) and pulsed-tissue Doppler-derived *e*′ velocity of the septal mitral annulus. Right ventricular (RV) end-diastolic basal diameter, tricuspid annular plane systolic excursion (TAPSE), Tissue Doppler *S*′ peak velocity, fractional area change, and tricuspid regurgitation peak velocity (TRV) were assessed in the RV-focused apical four chamber view (4, 9, 15, 16). RV systolic pressure was computed from TRV with the simplified Bernoulli equation (4, 9).

IVC diameter was measured in the subcostal view just proximal to the junction of the HV, at end-expiration and then end-inspiration to determine the respiratory variation (4–6, 9). HV flow was evaluated by PW Doppler in the subcostal view. Peak systolic and diastolic wave velocities (Vs and Vd, respectively) and the relevant velocity-time intervals (VTIs and VTId) were measured, and then the HV systolic filling fraction (HVFF) was calculated as VTIs/(VTIs + VTId) (4, 7, 10). Tricuspid *E*/*e*′ ratio was derived by the tricuspid inflow E wave velocity (as determined by PW Doppler, with the sample volume at the tips of the leaflets during the RV-focused apical four-chamber view) and tricuspid lateral annulus *e*′ wave velocity (with tissue Doppler imaging) (4, 8, 9). As tricuspid inflow and HV flow are highly sensitive to the respiratory phase, measurements from multiple beats were averaged.

**Figure 1:**
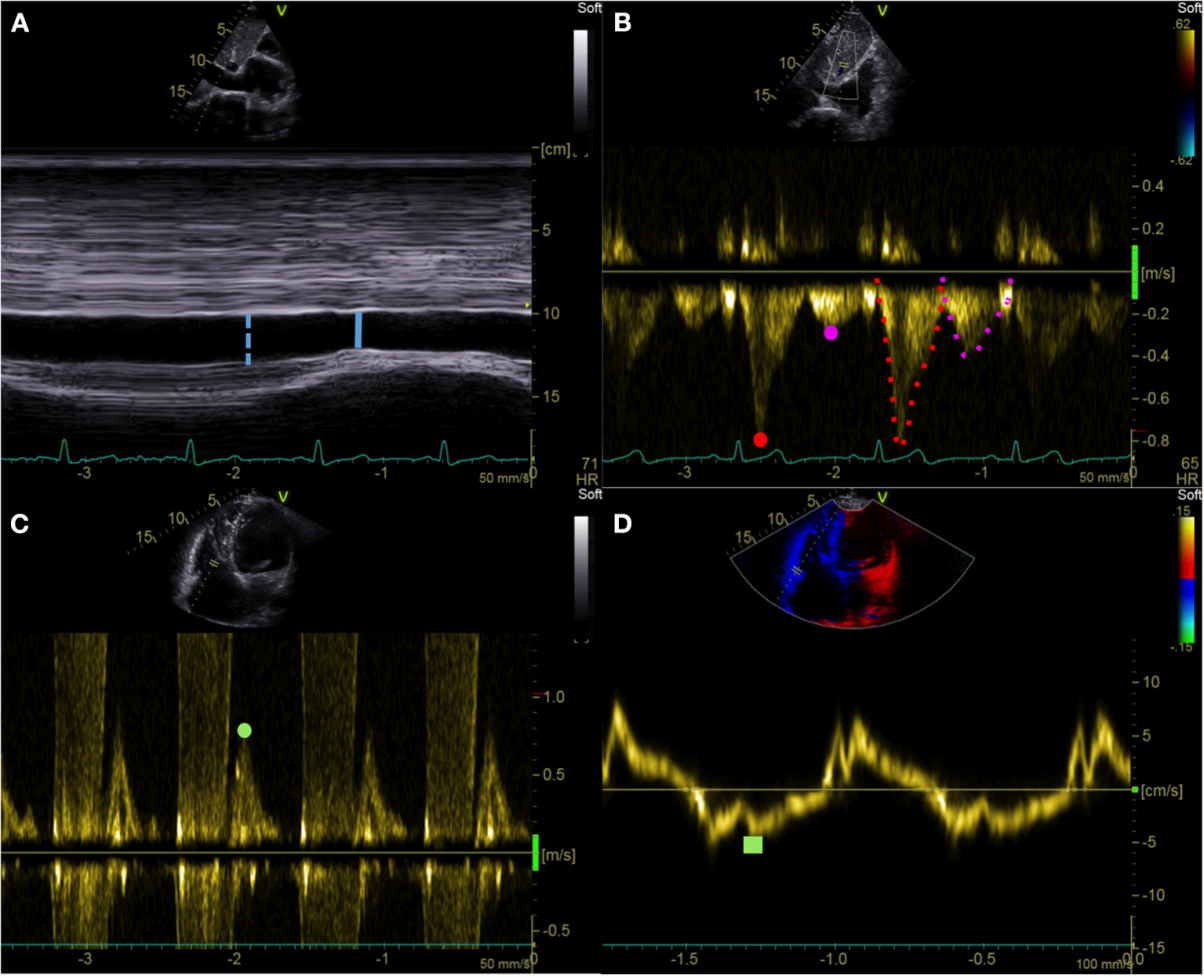
Echocardiographic right atrial pressure estimation. **(A)** IVC end-expiratory diameter (blue dotted line) and respiratory variation (solid line). **(B)** HV pulsed wave Doppler assessment: Vs (red dot), Vd (purple dot), VTIs (red dotted line), and VTId (purple dotted line). **(C,D)** Tricuspid *E*/*e*′ ratio: pulsed wave Doppler tricuspid inflow early E-wave peak velocity (green dot) and tricuspid lateral annulus tissue Doppler imaging *e*′ wave velocity (green square). IVC, inferior vena cava; HV, hepatic vein; Vs, hepatic vein peak systolic velocity; Vd, hepatic vein peak diastolic velocity; VTIs, velocity-time interval of the HV systolic wave; VTId, velocity-time interval of the HV diastolic wave.

eRAP_IVC_, ${\text{eRAP}}_{\text{E}/{\text{e}}^{\prime }}$, and eRAP_HV_ were given a value between 5 and 20 mmHg on a 5-mmHg scale as summarized in Table 1 and exemplified in Figure 1. eRAP_mean_ was calculated as (eRAP_IVC_ + ${\text{eRAP}}_{\text{E}/{\text{e}}^{\prime }}$ + eRAP_HV_)/3 and thereby consisted of continuous values.

**Table 1:** Scoring system of right atrial pressure as estimated by echocardiography.

**Assigned value**	**eRAP_**E/**e**'**_**	**eRAP_**HV**_**	**eRAP_**IVC**_**	**eRAP_**mean**_**
20 mmHg	> 8	Vs < Vd and HVFF <45% ORVs reverse	IVC >21 mm, no collapse	(${\text{eRAP}}_{E/e^{\prime }}$+ eRAP_HV_ + eRAP_IVC)_)/3 OR mean of available values
15 mmHg	6 < x ≤ 8	Vs < Vd and HVFF <55%	IVC >21 mm, <50% collapse	
10 mmHg	4 < *x* ≤ 6	Vs < Vd and HVFF >55%	IVC >21 mm, >50% collapse OR IVC ≤ 21 mm, <50% collapse	
5 mmHg	≤ 4	Vs > Vd	IVC ≤ 21 mm, ≥50% collapse	

*${\text{eRAP}}_{\text{E}/{\text{e}}^{\text{′}}}$, estimated right atrial pressure (eRAP) based on the tricuspid E/e′ ratio; eRAP_HV_, eRAP based on the hepatic vein (HV) pulsed wave Doppler spectra; eRAP_IVC_, eRAP based on inferior vena cava (IVC) diameter and respiratory variation; eRAP_mean_, mean of the different eRAP; Vs, HV peak systolic velocity; Vd, HV peak diastolic velocity; HVFF, HV systolic filling fraction*.

Interobserver variability was quantified by weighted κ analysis, with *k* values of < 0.21, 0.21–0.40, 0.41–0.60, 0.61–0.80, and 0.81–1 being considered poor, fair, moderate, good, and very good agreement, respectively (17).

### Right Heart Catheterization

RHC was performed under local anesthesia in the cardiac catheterization laboratory by other cardiologists (G.C., M.B., I.P., and P.A.), who were unaware of the results of the echocardiography. A balloon-tipped Swan-Ganz catheter was introduced through a sheath inserted into the femoral, antecubital, or jugular vein. The zero reference level was set at the midthoracic level. The catheter was advanced through the right heart chambers to the pulmonary artery, and pressures were measured. Then, the balloon was inflated, and the catheter was pushed forward up to the wedge position to record pulmonary artery wedge pressure. Finally, RV pressures and iRAP (mean over 5 cardiac cycles) were measured during catheter pull-back. Cardiac output was obtained by means of the thermodilution technique or Fick's indirect method (Dehmer formula), with the latter one being preferred in the presence of intracardiac or extracardiac shunts or severe tricuspid regurgitation.

### Statistical Analysis

Statistical analyses were performed using IBM SPSS Statistics version 25.0. GraphPad Prism was also used to make the Figures.

Normality was assessed with the Kolmogorov–Smirnov test. Continuous variables are presented as mean ± standard deviation or median with interquartile range, as appropriate. Categorical variables are reported as absolute count and percentages. The relationship between iRAP and IVC diameter, tricuspid *E*/*e*′ ratio, HVFF, or eRAP_mean_ was analyzed by Spearman correlation ρ test. The correlation between eRAP_mean_ and iRAP was also visually appraised by the Bland–Altman method. Furthermore, the correspondence between iRAP and eRAP_IVC_, ${\text{eRAP}}_{\text{E}/{\text{e}}^{\prime }}$, and eRAP_HV_ values was evaluated using the Wilcoxon signed-rank test. The 5- and 10-mmHg eRAP thresholds were tested against the same iRAP thresholds by receiver operating characteristic (ROC) areas under the curve (AUCs), and the eRAP_HV_, ${\text{eRAP}}_{\text{E}/{\text{e}}^{\prime }}$, and eRAP_mean_ AUC were compared with the eRAP_IVC_ AUC by means of the Wald test.

Because eRAP_mean_ and its components had not been compared before, no expected difference between these assessments was available to set a minimum number of enrolled patients.

## Results

Forty-three patients were included in the analysis. Their characteristics are shown in Table 2. The reasons for RHC were PH diagnosis (29 subjects, of whom 6 were found with PAH, 5 with chronic thromboembolic PH, and 4 with left heart disease–associated PH, and 14 did not actually have PH), PAH reassessment (10 subjects), or evaluation of HF eligibility to LVAD or heart transplant (4 subjects). Median age was 69 (58–75) years, and 28 (65%) patients were older than 65 years; male and female genders were equally distributed. Functional class was most often II, and median N-terminal pro–brain natriuretic peptide was 462 (114–2,045) ng/L. At the hemodynamic evaluation, median iRAP was 7 (3–11) mmHg, and 67% of the patients had an iRAP value below the 8-mmHg cutoff that identifies a higher risk of mortality (1, 18).

**Table 2:** Clinical, echocardiographic and hemodynamic characteristics of the study population.

**Age (years)**	**69 [58–75]**
Males	21 (49)
NYHA class I	4 (9)
II	24 (56)
III	11 (26)
IV	4 (9)
Systolic blood pressure (mmHg)	120 [105–140]
Heart rate (beat/min)	70 [65–84]
NT-proBNP (ng/L)	462 [114–2045]
Hemoglobin (g/dl)	12.8 ± 1.9
Creatinine (mg/dl)	1.0 [0.8–1.3]
β-Blocker	19 (44)
RASi	17 (49)
Loop diuretic	23 (54)
PAH therapy	11 (26)
RV basal diameter (mm)	40 ± 9
TAPSE (mm)	19 ± 4
RV FAC (%)	32 ± 11
RV *S*′ peak velocity (m/s)	0.12 ± 0.03
TRV (m/s)	3.4 ± 0.8
RVSP (mmHg)	51 ± 23
IVC diameter at end-expiration (mm)	16 (12–20)
Hepatic Vs (m/s)	0.4 ± 0.6
Hepatic Vd (m/s)	0.5 ± 0.3
Hepatic Vs/Vd ratio	1.4 ± 0.6
HVFF	53 ± 14
Tricuspid *E*/*e*′ ratio	4.1 [3.5–5.5]
Mitral E/A	0.9 [0.7–1.2]
Mitral *E*/*e*′ ratio	9 ± 4
Mitral DT E (ms)	211 ± 55
LVEF <55%	6 (14)
sPAP (mmHg)	53 ± 22
dPAP (mmHg)	20 ± 10
mPAP (mmHg)	32 ± 13
PAWP (mmHg)	11 ± 7
iRAP (mmHg)	7 [3–11]
Cardiac index (L/min per m^2^)	2.8 [2.4–3.5]
PVR (WU)	3 [1.3–7]

Data are presented as mean ± SD, median [IQR] or n (%), as appropriate.

*NYHA, New York Heart Association functional class; AF, atrial fibrillation; NT-proBNP, N-terminal pro brain natriuretic peptide; RASi, renin-angiotensin system inhibitors; PAH, pulmonary arterial hypertension; RV, right ventricular; TAPSE, tricuspid annular plane systolic excursion; FAC, fractional area change; TRV, tricuspid regurgitation peak velocity; RVSP, right ventricular systolic pressure; IVC, inferior vena cava; Vs, hepatic vein peak systolic velocity; Vd, hepatic vein peak diastolic velocity; HVFF, hepatic vein filling fraction; DT, deceleration time; LVEF, left ventricular ejection fraction; sPAP, systolic pulmonary arterial pressure; dPAP, diastolic pulmonary arterial pressure; mPAP, mean pulmonary arterial pressure; PAWP, pulmonary artery wedge pressure; iRAP, invasive right atrial pressure; PVR, pulmonary vascular resistance*.

Echocardiographic assessment of IVC was feasible in the entire study population, whereas HV parameters and tricuspid *E*/*e*′ ratio were not determinable in 4 and 2 patients, respectively. Interobserver agreement was very good (weighted *k* = 0.84, 0.90, and 0.87 for eRAP_IVC_, ${\text{eRAP}}_{\text{E}/{\text{e}}^{\prime }}$, eRAP_HV_, respectively). Median eRAP_IVC_, ${\text{eRAP}}_{\text{E}/{\text{e}}^{\prime }}$, eRAP_HV_, and eRAP_mean_ were 5 (5–10), 5 (5–20), 10 (5–10), and 6.7 (5–11.7) mmHg, respectively.

**Figure 2:**
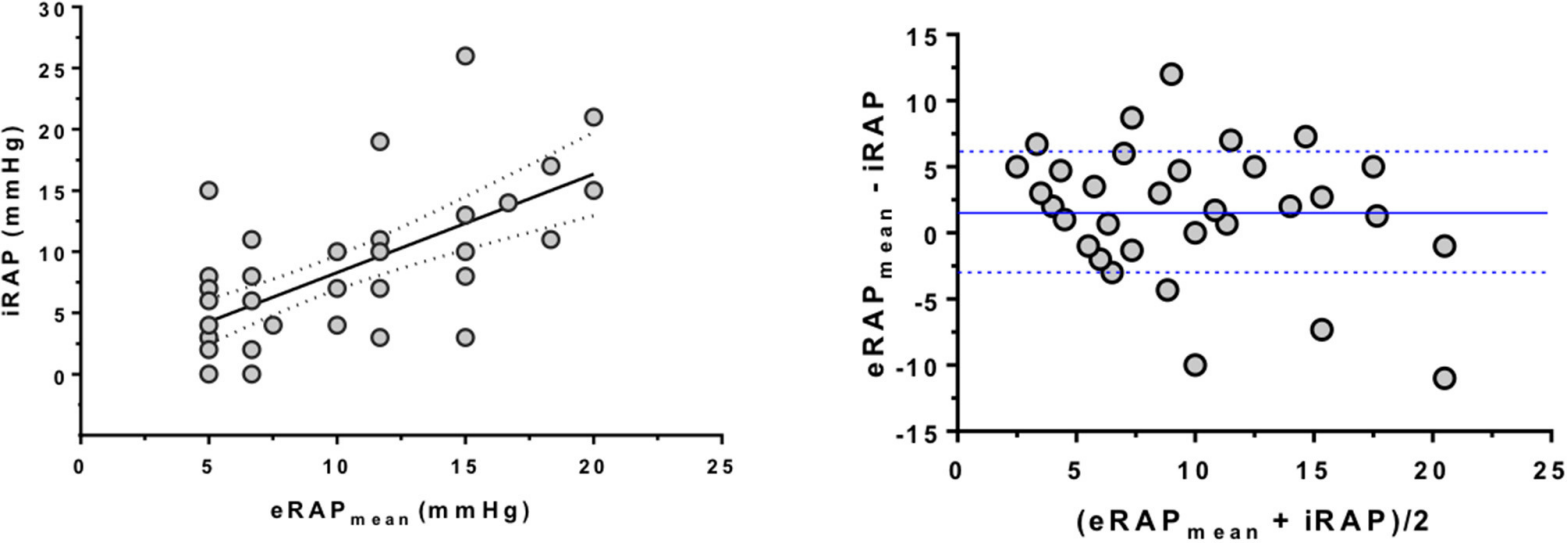
Correlation between multiparametric estimation and invasive measurement of right atrial pressure. (**Left**) Positive correlation between eRAP_mean_ and iRAP as assessed by RHC (Spearman correlation test). (**Right**) Bland–Altman plot showing that estimation of iRAP by eRAP_mean_ was especially good for values <10 mmHg. The blue lines represent the average ± 1 standard deviation of (eRAP_mean_ and iRAP). Note that in both analyses some subjects had the same values, hence the relevant dots overlap in the graphs. eRAP_mean_, multiparametric estimated RAP; iRAP, invasive RAP; RHC, right heart catheterization.

**Figure 3:**
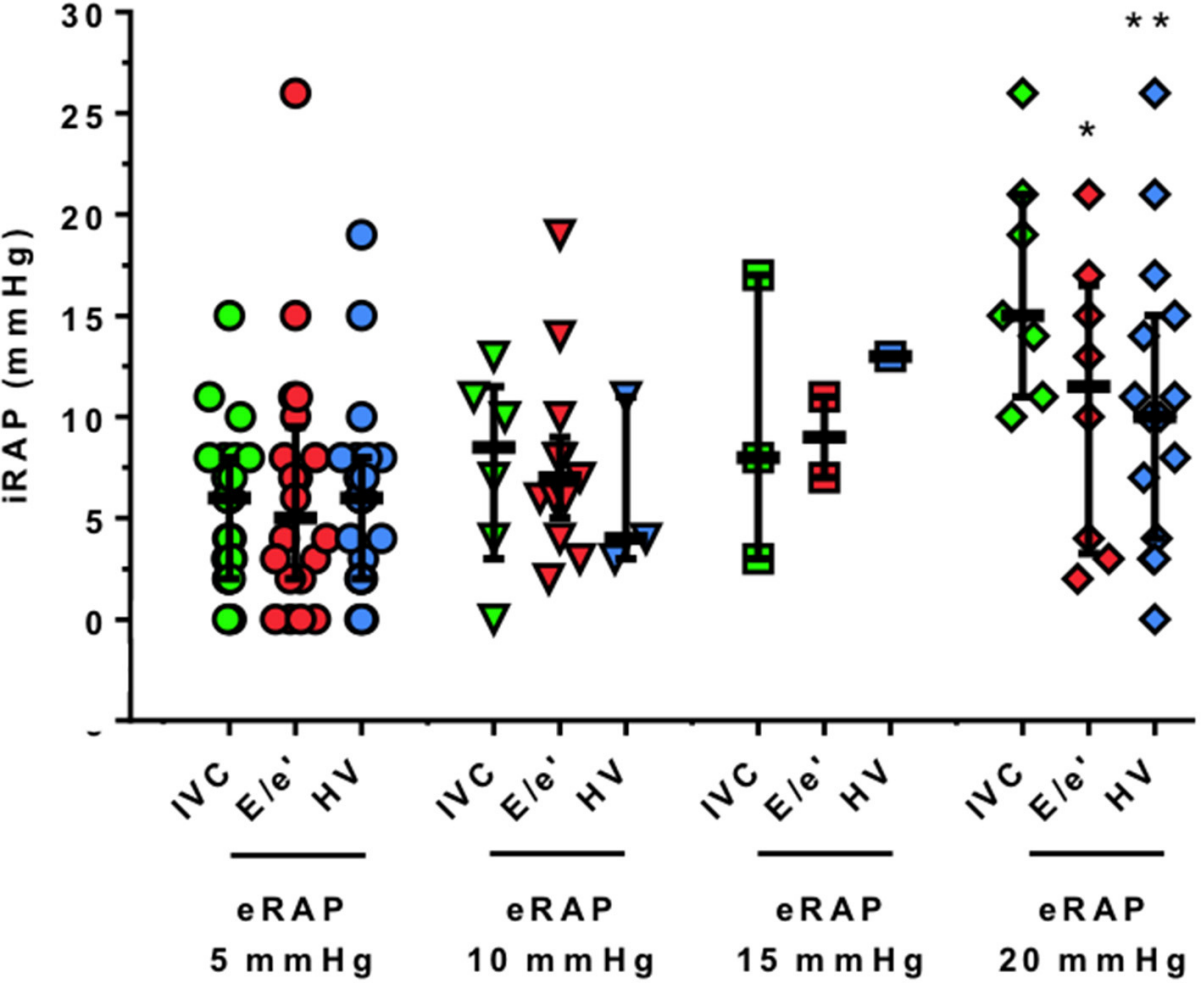
Correlation between single-parameter estimation and invasive measurement of right atrial pressure. The actual values of iRAP obtained during RHC are presented for each 5-mmHg threshold and by eRAP component. Horizontal bars indicate medians and interquartile ranges, *P < 0.05 and **P < 0.001, respectively (Wilcoxon signed-rank test). iRAP, invasive right atrial pressure; eRAP, estimated right atrial pressure; IVC, inferior vena cava; E/e′ ratio of pulsed wave Doppler tricuspid inflow early E-wave peak velocity and tricuspid lateral annulus tissue Doppler imaging e′ wave velocity; HV, hepatic veins.

The parameters from which eRAP_IVC_, ${\text{eRAP}}_{\text{E}/{\text{e}}^{\prime }}$, and eRAP_HV_ are derived were positively correlated with iRAP: *r* was 0.47 for IVC diameter (*P* = 0.002), 0.44 for tricuspid *E*/*e*′ ratio (*P* = 0.004), and 0.46 for HVFF (*P* = 0.007). Consistently, there was also a positive correlation between eRAP_mean_ and iRAP (*r* = 0.66, *P* < 0.001; Figure 2, **left**). The Bland–Altman plot showed that eRAP_mean_ was in agreement with iRAP especially when ≤ 10 mmHg (Figure 2, **right**). For all eRAP components, 5 mmHg was the most frequent estimate, and the actual iRAP was not significantly different from it (Figure 3). For the 10-mmHg category, the concordance between eRAP components and iRAP was less frequent, particularly for ${\text{eRAP}}_{\text{E}/{\text{e}}^{\prime }}$ and eRAP_HV_, although not to a statistically significant extent. For the 15-mmHg value, it was possible to test only the correlation between iRAP and eRAP_IVC_ (no significant difference), as the number of ${\text{eRAP}}_{\text{E}/{\text{e}}^{\prime }}$ and eRAP_HV_ was too low. A statistically significant difference between iRAP and ${\text{eRAP}}_{\text{E}/{\text{e}}^{\prime }}$ (*P* = 0.02) and eRAP_HV_ (*P* < 0.001) was instead found for the 20-mmHg threshold (Figure 3).

**Figure 4:**
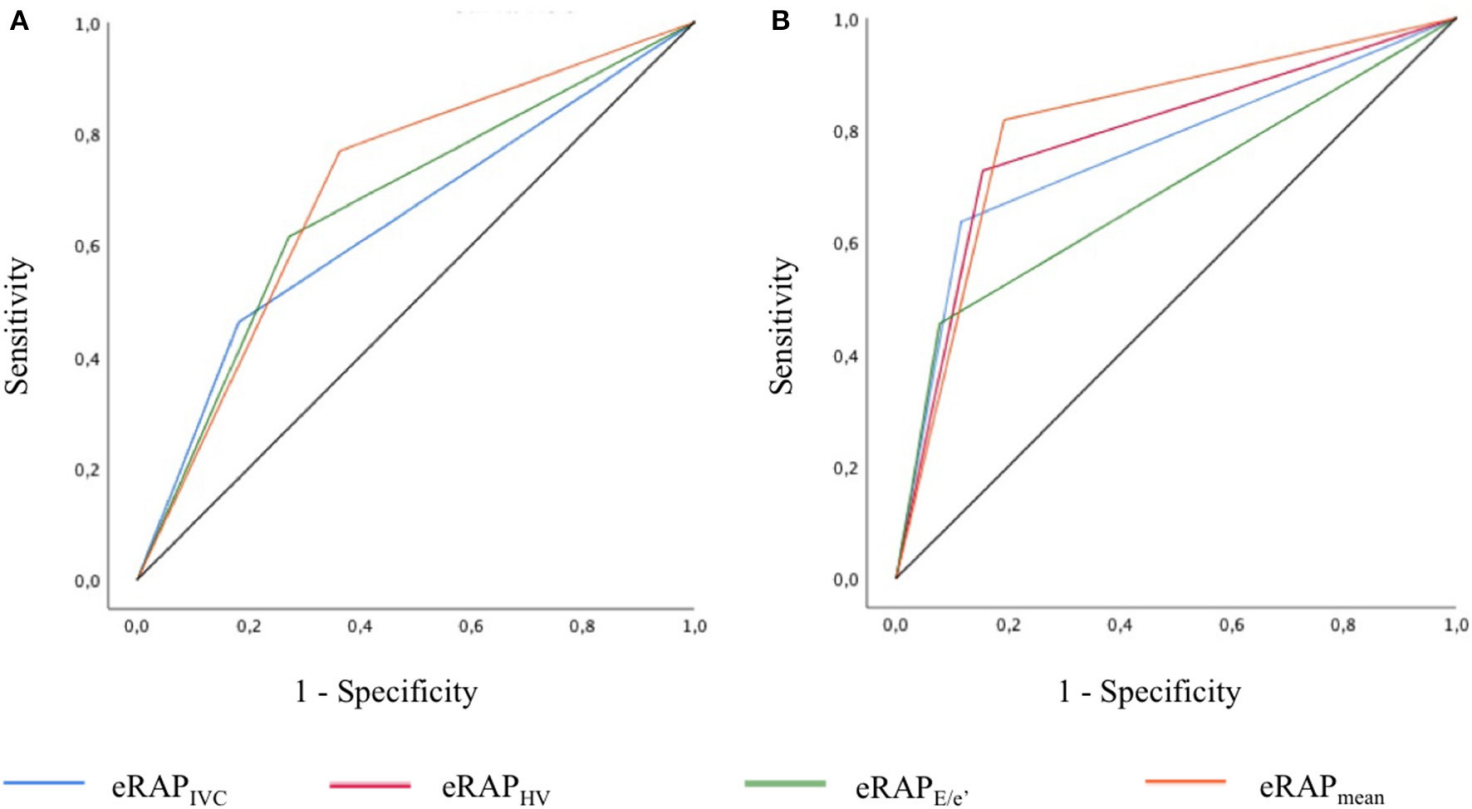
ROC curves showing the accuracy of the different modalities of estimation of right atrial pressure in predicting the actual value, as measured during right heart catheterization, for the 5-mmHg **(A)** and 10-mmHg **(B)** thresholds. In **(A)**, the AUC of eRAP_IVC_ and eRAP_HV_ overlap. eRAP_IVC_, estimated right atrial pressure (eRAP) based on inferior vena cava (IVC) diameter and respiratory variation; *eRAP*_*HV*_, eRAP based on the hepatic vein (HV) pulsed wave Doppler spectra; eRAP_E/*e'*_, eRAP based on the tricuspid *E*/*e*′ ratio; eRAP_mean_, mean of the different eRAP.

The accuracy in predicting iRAP was numerically highest for eRAP_mean_ for both the 5- and the 10-mmHg categories (Table 3 and Figure 4). Nonetheless, the AUC of eRAP_mean_ was not significantly different from that of eRAP_IVC_, nor were the AUC of eRAP_HV_ and ${\text{eRAP}}_{\text{E}/{\text{e}}^{\prime }}$ (Table 3).

**Table 3:** Accuracy of the different methods for estimating right atrial pressure.

	**iRAP** **>5 mmHg**			**Contrast *P*-value**
	**AUC**	**95% CI**	**Standard error**	
eRAP_IVC_	0.64	(0.49–0.80)	0.08	-
eRAP_HV_	0.64	(0.49–0.80)	0.08	1.00
${\text{eRAP}}_{E/e^{\prime }}$	0.67	(0.50–0.84)	0.09	0.75
eRAP_mean_	0.70	(0.53–0.87)	0.09	0.41
	**iRAP** **>10 mmHg**			**Contrast** ***P*****-value**
	**AUC**	**95% CI**	**Standard error**	
eRAP_IVC_	0.76	(0.60–0.92)	0.08	—
eRAP_HV_	0.79	(0.63–0.94)	0.08	0.78
${\text{eRAP}}_{E/e^{\prime }}$	0.69	(0.53–0.85)	0.08	0.46
eRAP_mean_	0.81	(0.67–0.96)	0.07	0.43

For both the 5- and the 10-mmHg threshold, the ROC AUC of eRAP_HV_, ${\text{eRAP}}_{\text{E}/{\text{e}}^{\text{′}}}$, and eRAP_mean_ were compared with the one of eRAP_IVC_: the resulting P-values are shown in the right column.

*AUC, area under the curve; eRAP_IVC_, estimated right atrial pressure (eRAP) based on inferior vena cava (IVC) diameter and respiratory variation; eRAP_HV_, eRAP based on the hepatic vein (HV) pulsed wave Doppler spectra; ${\text{eRAP}}_{\text{E}/{\text{e}}^{\text{′}}}$, eRAP based on the tricuspid E/e′ ratio; eRAP_mean_, mean of the different eRAP; iRAP, invasive right atrial pressure*.

## Discussion

eRAP is part of the standard transthoracic echocardiographic examination and provides important information. It is fundamental in the diagnostic workup of PH, as systolic pulmonary artery pressure is calculated as the sum of eRAP and RV systolic pressure (1, 4, 9). Furthermore, elevated eRAP is associated with worse prognosis in HF (19, 20) and PAH (21).

In clinical practice, eRAP is obtained by examining the dimension and respiratory collapsibility of IVC (6). Other methods for eRAP exist, but have not been validated across different populations (11). Hence, eRAP_IVC_ is recommended as the default approach, with other modalities being complementary (4, 22). Nonetheless, eRAP_IVC_ is approximate. A semiautomated assessment of IVC collapsibility and pulsatility has recently been proposed to overcome the limitations of eRAP_IVC_ (23). Alternatively, eRAP could be more precise if the estimates attained with different techniques were incorporated into a multiparametric scoring system (13).

On this background, we determined the accuracy of averaging the values of eRAP derived from the evaluation of IVC, HV PW Doppler profiles, and tricuspid *E*/*e*′ ratio. Although eRAP_mean_ did correlate with iRAP, it did not perform significantly better than eRAP_IVC_ in predicting iRAP.

Individual comparisons of eRAP_IVC_, ${\text{eRAP}}_{\text{E}/{\text{e}}^{\prime }}$, and eRAP_HV_ with iRAP have already been drawn (6–8, 24–27). By contrast, to our knowledge, only one recent investigation with LVAD patients considered eRAP_IVC_, ${\text{eRAP}}_{\text{E}/{\text{e}}^{\prime }}$, and eRAP_HV_ together to compute eRAP_mean_ (14). In this study like in ours, eRAP_mean_ had the greatest AUC for the detection of iRAP >10 mmHg. However, the authors focused on the value of eRAP_mean_ in combination with several other echocardiographic variables in guiding LVAD management, and no statistical comparison between ROC was performed, precluding any conclusion about the higher accurateness of eRAP_mean_ over eRAP_IVC_. It is also notable that we included a heterogeneous cohort of subjects, a crucial step to understand the potential clinical value of eRAP_mean_.

For each eRAP_mean_ component (eRAP_IVC_, ${\text{eRAP}}_{\text{E}/{\text{e}}^{\prime }}$, and eRAP_HV_), echocardiographic and invasive values were more often similar when eRAP was <10 mmHg. As a consequence, the correspondence between eRAP_mean_ and iRAP also appeared to be looser for eRAP_mean_ values >10 mmHg. The highest discordance with iRAP was found for ${\text{eRAP}}_{\text{E}/{\text{e}}^{\prime }}$ and eRAP_HV_ >10 mmHg. Consistent with our results, the cutoffs beyond which ${\text{eRAP}}_{\text{E}/{\text{e}}^{\prime }}$ was less reliable in previous studies were also <10 mmHg (8, 28).

Overall, the present work supports the systematic use of eRAP_IVC_ in the clinical arena, as it is the simplest way to estimate RAP. Moreover, an extensive literature indicates that the echocardiographic evaluation of IVC offers diagnostic and prognostic cues *per se*, regardless of which value is assigned to eRAP_IVC_. Demonstration of a dilated and/or non-collapsible IVC may be sufficient to identify patients with HF and increased LV filling pressures (29) and has been associated with HF hospitalization and mortality (19, 30, 31). In addition, a larger IVC size at discharge was related to a higher risk of readmission after a first hospitalization for HF (32, 33). An independent prognostic role of IVC dilation and reduced collapsibility has also been shown in PAH (34). However, ${\text{eRAP}}_{\text{E}/{\text{e}}^{\prime }}$ and eRAP_HV_ may be more convenient in specific populations. ${\text{eRAP}}_{\text{E}/{\text{e}}^{\prime }}$ can be helpful in patients with a poor subcostal ultrasound window (24), and HVFF has specifically been evaluated in mechanically ventilated patients (7).

Until eRAP_IVC_ remains the reference in clinical practice, efforts to improve it are desirable, for instance, by tracking the respirophasic movements of the IVC in echocardiographic videoclips (23).

We acknowledge that the sample we examined was small and mostly made of subjects with a low iRAP. Thus, the data presented here should be viewed as preliminary to bigger studies with a wider range of iRAP. On the other hand, this work is the first one addressing the performance of eRAP_mean_ in a series of consecutive patients with different cardiac disorders. It is also remarkable that eRAP and iRAP were assessed on the same day and, in most cases, few hours apart by reciprocally blinded investigators.

## Conclusions

The optimal approach for eRAP during transthoracic echocardiography is debated; recently, it has been suggested that incorporating the analysis of IVC, tricuspid *E*/*e*′ ratio, and HV is better than relying only on IVC assessment.

In this prospective cohort of patients in whom RAP was invasively measured, however, multiparametric eRAP was not more precise than the estimate based on IVC, tricuspid *E*/*e*′ ratio, or HV.

While awaiting for additional studies, we conclude that, at present, evaluation of IVC diameter and collapsibility is preferable for eRAP.

## Data Availability Statement

The raw data supporting the conclusions of this article will be made available by the authors, without undue reservation.

## Ethics Statement

The studies involving human participants were reviewed and approved by IRCCS Ospedale Policlinico San Martino Institutional Review Board. The patients/participants provided their written informed consent to participate in this study.

## Author Contributions

MT and PA designed the study, collected and data, and wrote the article. SG, GC, GM, MB, and IP collected data. GC and MC analyzed data. FM wrote the article. All authors contributed to the article and approved the submitted version.

## Conflict of Interest

The authors declare that the research was conducted in the absence of any commercial or financial relationships that could be construed as a potential conflict of interest.
